# Whole genome sequencing of two human rhinovirus A types (A101 and A15) detected in Kenya, 2016-2018

**DOI:** 10.12688/wellcomeopenres.16911.1

**Published:** 2021-07-08

**Authors:** Martha M. Luka, Everlyn Kamau, Zaydah R. de Laurent, John Mwita Morobe, Leonard K. Alii, D. James Nokes, Charles N. Agoti

**Affiliations:** 1 Epidemiology and Demography Department, KEMRI-Wellcome Trust Research Programme, Centre for Geographic Medicine Research-Coast, Kilifi, Kenya; 2 Department of Biochemistry and Biotechnology, Pwani University, Kilifi, Kenya; 3 Department of Mathematics and Computer Science, Pwani University, Kilifi, Kenya; 4 School of Life Sciences and Zeeman Institute for Systems Biology and Infectious Disease Epidemiology Research (SBIDER), University of Warwick, Coventry, UK; 5 Department of Public Health, Pwani University, Kilifi, Kenya

**Keywords:** human rhinovirus, whole-genome, sequencing, phylogenetics

## Abstract

**Background:** Virus genome sequencing is increasingly utilized in epidemiological surveillance. Genomic data allows comprehensive evaluation of underlying viral diversity and epidemiology to inform control. For human rhinovirus (HRV), genomic amplification and sequencing is challenging due to numerous types, high genetic diversity and inadequate reference sequences.

**Methods:** We developed a tiled amplicon type-specific protocol for genome amplification and sequencing on the Illumina MiSeq platform of two HRV types, A15 and A101. We then assessed added value in analyzing whole genomes relative to the VP4/2 region only in the investigation of HRV molecular epidemiology within the community in Kilifi, coastal Kenya.

**Results: **We processed 73 samples collected between 2016-2018, and 48 yielded at least 70% HRV genome coverage. These included all A101 samples (n=10) and 38 (60.3%) A15 samples.  Phylogenetic analysis revealed that the Kilifi A101 sequences interspersed with global A101 genomes available in GenBank collected between 1999-2016. On the other hand, our A15 sequences formed a monophyletic group separate from the global genomes collected in 2008 and 2019. Improved phylogenetic resolution was observed with the genome phylogenies compared to the VP4/2 phylogenies.

**Conclusions: **We present a type-specific full genome sequencing approach for obtaining HRV genomic data and characterizing infections.

## Introduction

Genomic surveillance of respiratory viruses is important for (i) developing molecular diagnostics ^1, 2^, (ii) investigating transmission and evolution ^3, 4^, and (iii) development of vaccines and therapeutic drugs ^5^. Human rhinovirus (HRV) is the most common cause of upper respiratory infections ^6, 7^ and is also occasionally associated with lower respiratory infections ^8^. It is a highly diverse virus, with over 160 distinct types identified globally ^9^. This diversity presents a challenge in developing a sequencing protocol that works well across the different HRV types ^10^. Most HRV molecular epidemiology studies utilize partial genome sequences, which offer lower resolution in identifying epidemiologically linked infections ^11^.

Viral genome sequencing can take one of the two approaches available: a targeted/enrichment approach or an agnostic/metagenomics approach ^12^. Theoretically, the viral metagenomics approach is an unbiased way to obtain all viral genetic content in a sample as it does not require prior knowledge of their sequences ^13^. However, this approach requires high viral titers to succeed ^14^, which are not always available in a clinical sample ^15^. Furthermore, most clinical samples especially those relevant to HRV sequencing are dominated by host and bacterial nucleic acids ^13^. Nonetheless, this challenge can be overcome by target enrichment, for example, polymerase chain reaction (PCR) to bulk up for the target virus before sequencing ^15– 17^. We describe a target enrichment sequencing approach of two HRV types, A15 and A101, using type-specific primers and compare the phylogenetic inferences between partial and whole genome sequences.

## Methods

### Study population

The study utilized nasopharyngeal swabs collected during two previous studies in Kilifi County, coastal Kenya: outpatient surveillance of nine health dispensaries within the Kilifi Health and Demographic Surveillance System (KHDSS) between January and December 2016 ^7^ and primary school surveillance between May 2017 and April 2018 ^6^. The school was situated in Junju, a location within the KHDSS. All samples were collected from symptomatic individuals (mild symptoms of acute respiratory tract infection) of varied age (one month - 49 years) and archived at -80°C.

### Study design

Samples were screened for HRV and typed as previously described ^8^. A cycle threshold (Ct) value < 35.0 was used to define positives. HRV positives underwent VP4/2 sequencing to characterize the diversity, spatial and temporal occurrence of HRV in the two settings. Comparison of the HRV diversity within the school in Junju and the health dispensary located in Junju revealed 12 common types, and the most frequent common type was HRV-A101 (n=5 in each setting) ^9, 11^.

For this study, we purposively selected two types from the two studies: the most frequent type observed in the KHDSS health dispensary surveillance (A15, n=63) and the most frequent common type identified at the Junju health dispensary and school (A101, n=10) for whole genome sequencing (WGS).

### Ethics statement

Sample collection was undertaken following an informed written consent provided by parents or guardians for persons <18 years or by participating individuals if aged >17 years. Children whose parents consented were also asked for individual assent to participate. The study protocols were reviewed and approved by the University of Warwick Biomedical and Scientific Research Ethics Committee (BSREC #REGO-2016-1858 and #REGO-2015-6102) and the KEMRI-Scientific Ethics Review Unit (KEMRI-SERU #3332 and #3103).

### Primer design

We retrieved nine type A101 genomes and three type A15 genomes, all >6000 nt long from GenBank ^18^ on 30 ^th^ September 2019. Geneious Prime 2019.2.1 ( https://www.geneious.com) was used to design eight overlapping primer pairs across the ~7.2 kb HRV genome. The primers targeted eight amplicons 0.9–1.6 kb in size, with overlaps varying in size from 300 to 800 bases, Table 1.

**Table 1.** Type-specific primers for the whole-genome amplification of two human rhinovirus types-A15 and A101.

Name	Target type	Amplicon	Start	Length	%GC	Tm (°C)	Hairpin Tm (°C)	Self- dimer Tm (°C)	Pair dimer Tm (°C)	Sequence
95 F_a101	A101	1	95	23	39.1	58	None	None	6.9	ACCCCAAATGTAACTTAGAAGCA
716 R_a101	A101	1	1,334	22	45.5	60	None	None	None	TCATCAGTGGGTTGTTGTGAGT
726 F_a101	A101	2	726	20	50	60	None	None	None	AGCATCAAGTGGAGCGTCAA
1,215R_a101	A101	2	1,833	22	54.5	62	None	None	None	GACACCCACACGAACTGCATAC
827 F_a101	A101	3	1,445	22	36.4	56	None	None	None	ATGCTGTTCCTATGGATTCAAT
2,468R_a101	A101	3	2,468	20	50	60	None	None	None	TCTGGTTGTGTTTGGCTGGT
1,524F_a101	A101	4	2,142	22	40.9	56	None	None	None	TACCACACCTGATACATACTCA
3,516R_a101	A101	4	3,516	20	55	60	None	8.8	3.2	TCCACAATCTCCAGGTGCAC
2,546F_a101	A101	5	3,164	23	39.1	56	None	None	None	TACCTACAAGAACAGACCTTACT
3,901R_a101	A101	5	4,519	22	40.9	55	None	None	None	GTTTCCCTTTGTCTGGTAAATC
4,102F_a101	A101	6	4,102	20	50	60	None	None	None	ACCCAGAAACAGCAGCAAGA
5,248R_a101	A101	6	5,248	23	39.1	58	None	None	None	ACCCTGTGAACTTTCCATTACAT
4,306F_a101	A101	7	4,924	24	37.5	57	None	None	None	AAATCAGTTAGGAATCCAGATGTC
5,905R_a101	A101	7	6,523	24	33.3	56	None	None	None	TAGAATTACACAACTTCCTAACCA
5,550F_a101	A101	8	6,168	21	38.1	55	None	None	None	ACCAATGATCACTTTCCTCAA
6,383R_a101	A101	8	7,001	24	33.3	56	None	None	None	TGGTCATATTTGTCTTTTCCACTA
A15_1F	A15	1	21	20	55	61	None	None	None	ATCCCACCTGAACCTCCCAA
A15_1R	A15	1	1,251	20	55	60	None	None	None	CCAGCCGTGACATTACCTYT
534F_a15_22	A15	2	621	21	52.4	60	None	2.9	None	CCATGGGCGCTCAAGTATCTA
1,889R_a15_22	A15	2	1,988	24	33.3	57	None	None	None	CACAAAACATGAAACTGAATCGTA
1,391F_a15_22	A15	3	1,478	21	42.9	56	None	None	None	AGACATAACAACTGGAGCTTG
2,848R_a15_22	A15	3	2,947	23	34.8	56	None	None	None	TCCATCGTATCCATCATAAAACA
2,417F_a15_22	A15	4	2,516	22	40.9	55	None	None	None	TCACAGACTAGAGATGAGATGA
3,464R_a15_22	A15	4	3,563	20	45	55	None	None	None	CTATCACACCATGTTTGCAC
2,900F_a15_22	A15	5	2,999	24	33.3	54	None	4.3	None	CTATGTTCAAGAATAGTCACTGAA
4,352R_a15_22	A15	5	4,451	23	39.1	58	None	3	None	CACCAGGATTTTGCATAATGTCA
3,576F_a15_22	A15	6	3,675	21	38.1	55	None	None	None	TTGGTGACGGGTTTGTAAATA
4,991R_a15_22	A15	6	5,090	24	33.3	55	None	None	None	CAAATATAATGCCTGCTATACTGA
4,385F_a15_22	A15	7	4,484	23	43.5	58	None	None	None	TCAAGTGTAACCTTTATCCCTCC
5,943R_a15_22	A15	7	6,042	23	43.5	59	None	None	None	GTTCCAAACACACTATCCTCCAA
A15_8F	A15	8	5,972	20	55	60	None	None	None	ACYCTTGAYATTGRCCCAGC
A15_8R	A15	8	7,029	20	55	60	None	None	None	CTCACACTGCGAATCCCCTT
5,560F_a15_22	A15	8	5,659	21	42.9	55	None	None	None	CATTCATGTTGGTGGTAATGG
7,076R_a15_22	A15	8	7,076	20	55	60	None	None	None	AAGGCGGGATATACAGTGCG

Tm - melting temperature Start - Genome position (of the whole genome template used) where the primer sequence starts GenBank sequences used in primer design were accession numbers: MN306051.1, DQ473493.1 and JN541268.1 for A15 and; KY460514.1, GQ415052.1, KY369891.1, KY189315.1, KY369897.1, KY369892.1, KY369889.1, JQ245965.1 and GQ415051.1 for A101.

### RNA extraction, reverse transcription and PCR

Viral RNA was extracted from 140 μl sample using QIAamp Viral RNA kit (Qiagen, USA) as per the manufacturer's recommendations. Reverse transcription was carried out using random hexamers and the Superscript III First-Strand Synthesis System (Invitrogen, United Kingdom). Genome-wide amplification using HRV-specific primers was done using the Q5 High-Fidelity 2X Master Mix (New England Biolabs, United Kingdom). PCR success was assessed by electrophoresing the products on a 1% agarose gel. Once suitable PCR conditions per amplicon were established, a duplex PCR of non-consecutive amplicons of similar conditions was set up (Protocol doi - dx.doi.org/10.17504/protocols.io.bukxnuxn).

### Sequencing

PCR products were purified with 1X AMPure XP beads (Beckman Coulter Inc.), quantified with Qubit dsDNA High Sensitivity Assay (Invitrogen, United Kingdom), pooled per sample and normalized to 0.2 ng/uL. Sequencing libraries were prepared using the Nextera XT Sample Preparation Kit (Illumina, CA) and sequencing performed on Illumina MiSeq platform (200 bp × 2) per sample.

### Sequence assembly

The raw reads were quality checked using FastQC v0.11.9 and trimmed (Phred score >30) using Trimmomatic v0.39 ^19^. HRV reads were identified by mapping to the respective reference strains ( https://www.picornaviridae.com/sg3/enterovirus/rv-a/rv-a_seqs.htm) and subsequently assembled into contigs using SPAdes v3.12.0. The contigs were checked for completeness and assembled to a consensus sequence using Sequencher v5.4.6 ( www.genecodes.com). We defined sequencing success as obtaining HRV reads covering at least 70% of the genome (>5040 bases). Sequencing depth was visualized using the deepTools ^20^ package.

### Sequence analysis

Sequences were aligned using MAFFT v7.271 ^21^. Recombination scans were done using RDP5 ^22 ^and visualized on SimPlot ^23^. Nucleotide substitutions across the genomes were visualized using a python script to examine genetic diversity across the genome. POPART ^24^ was used to construct haplotype networks using the Minimum Spanning Network model. The best-fitting model and maximum likelihood trees were inferred using IQ-TREE, v1.6.0 ^25^. Branch support for phylogenetic trees was assessed using bootstrapping of 1000 iterations. MegaX ^26^ was used to calculate mean pairwise distances, and the respective standard errors were assessed using 100 iterations.

Bayesian phylogeny was used to create time-structured phylogenetic trees using BEAST v.1.10.4 ^27^. BEAST was run with 200 million MCMC steps using the best fitting substitution model and a coalescent-based relaxed clock framework ^28^. The output was assessed for convergence using Tracer v1.7.1. Maximum clade credibility (MCC) trees were identified using TreeAnnotator v1.10.4 after removal of 10% burn-in. The trees were then visualised in FigTree v1.4.4 ^29^ and branching posterior probabilities were noted.

### Statistical analysis

Statistical analysis was undertaken using R version 3.6.1 (R Core, 2021). The Shapiro–Wilk test was used to check for the normality of the data. The *T*-test was then used to compare Ct-values of successfully sequenced versus failed samples.

## Results

### Whole genome sequencing

**Figure 1.**
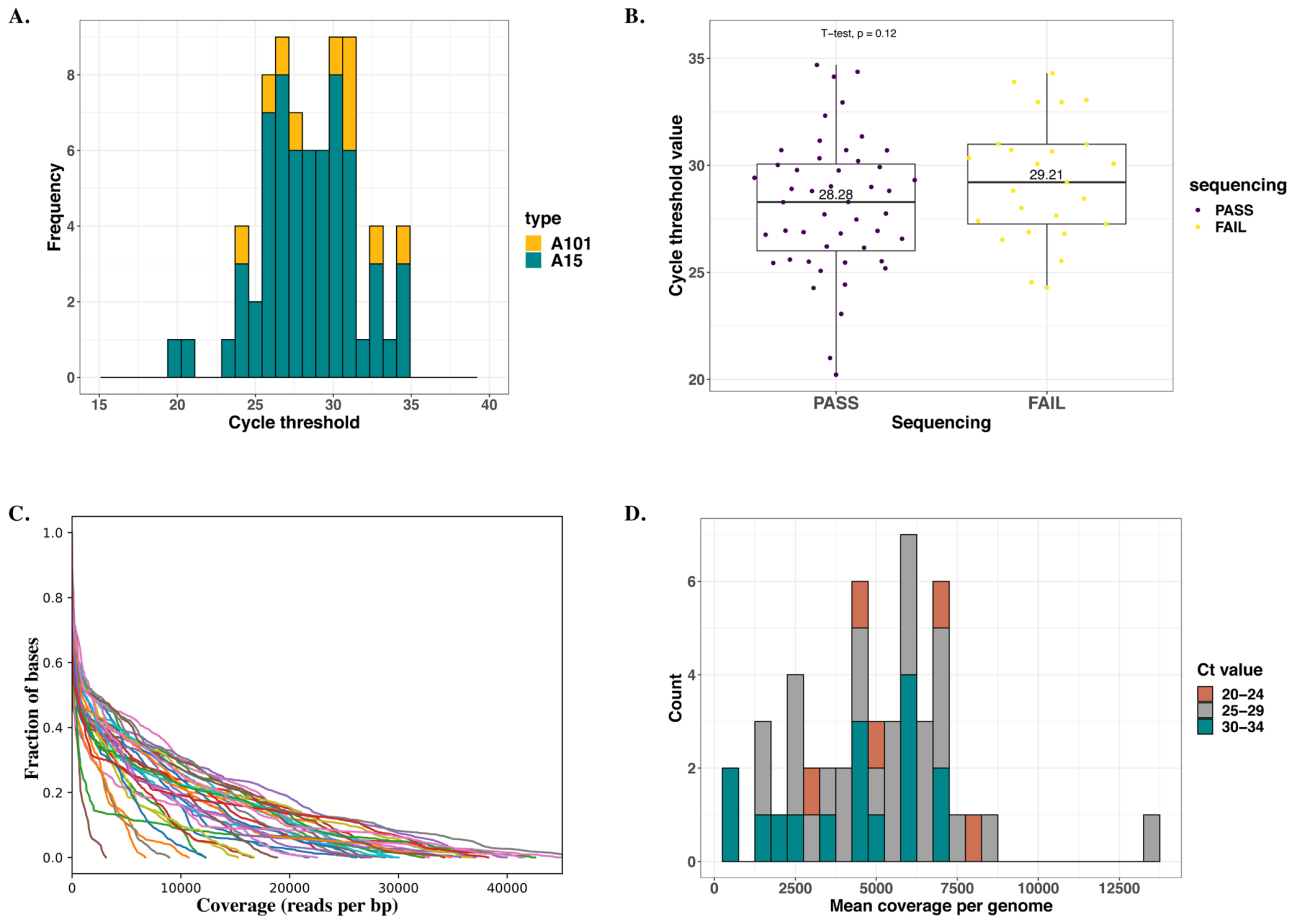
Summary statistics of sequenced samples. ( **A**) Distribution of cycle threshold (Ct) values across all samples selected for sequencing. Bars are colored by HRV type. ( **B**) Dispersion of Ct-values across samples successfully sequenced and those that failed. ( **C**). Read depth (per base pair) distribution per (successfully) sequenced sample. Each line represents a genome/sample. ( **D**). Distribution of mean coverage per base pair per genome across successfully sequenced samples. The bars are colored by Ct-value group.

We successfully sequenced all 10 (100%) A101 and 38 of 63 (60.3%) A15 samples. Cycle threshold (Ct) values ranged from 20.2 – 34.7, with a median of 28.4 for A15 and 30.2 for A101. The failed 25 samples did not have a significantly higher median Ct-value than those successfully sequenced based on the *T*-test: 28.3 (IQR = 4.0) versus 29.2 (IQR = 3.7), respectively *(p= 0.21*), Figure 1A and B. Besides, samples that failed sequencing did not have unique phylogenetic clustering patterns based on their previously generated VP4/2 sequences. Sequencing depth was comparable across Ct-values, with the mean depth coverage per genome ranging from 351 - 13356 reads per base pair, Figure 1C and D.

**Figure 2.**
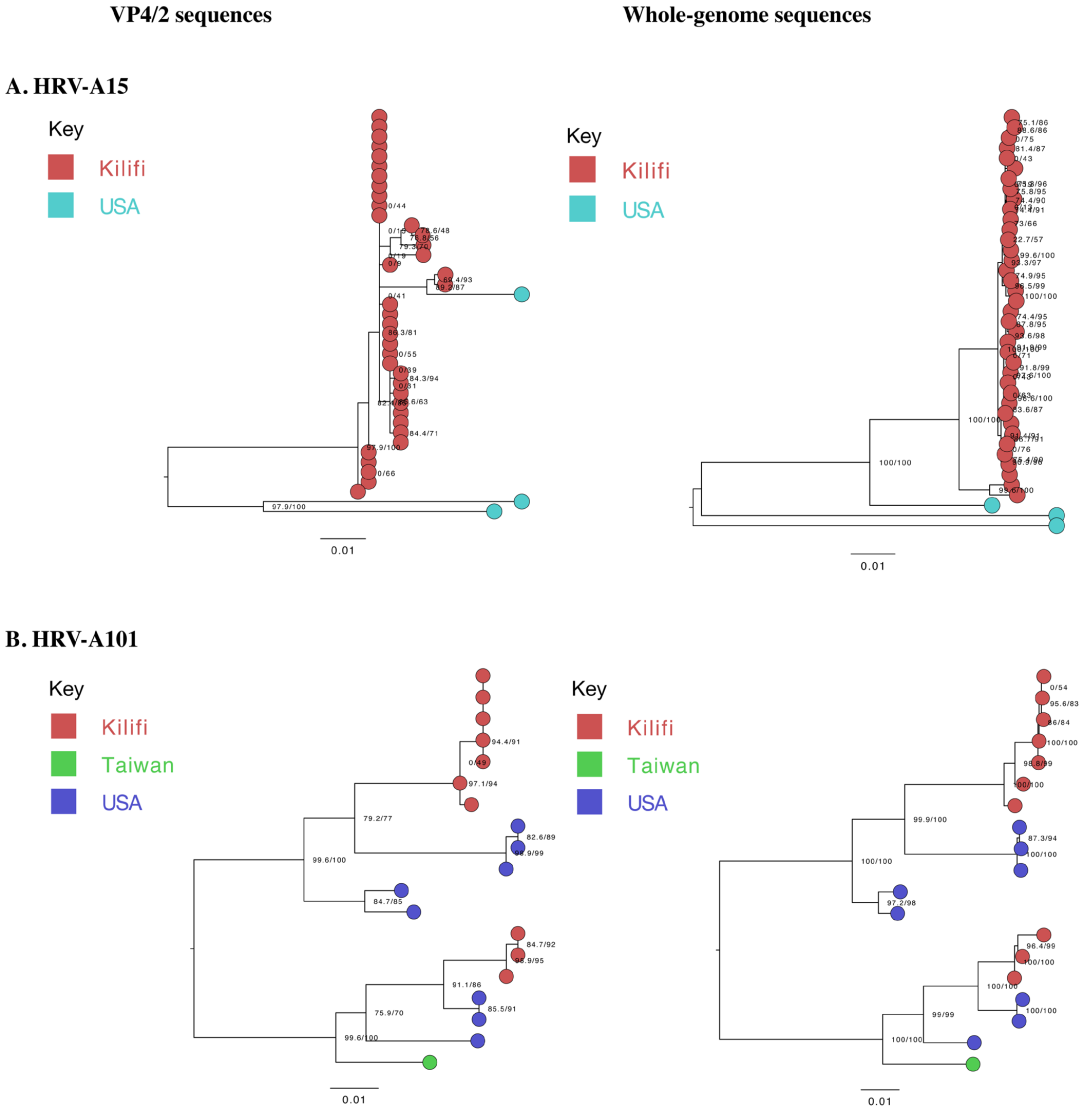
Maximum-likelihood phylogenetic trees of local (generated) and global ( **A**) A15 and ( **B**) A101 sequences. The tips are coloured by origin, indicating the global sequences used in primer design. The scale bar represents nucleotide substitutions per site.

Phylogenetic analysis identified interspersion of local A101 sequences with global sequences (n=9) collected between the years 1999–2016. However, A15 local genomes clustered separately from global sequences (n=3), Figure 2. The global A15 genomes were collected in the years 2008 (n=2) and 2019 (n=1).

**Figure 3.**
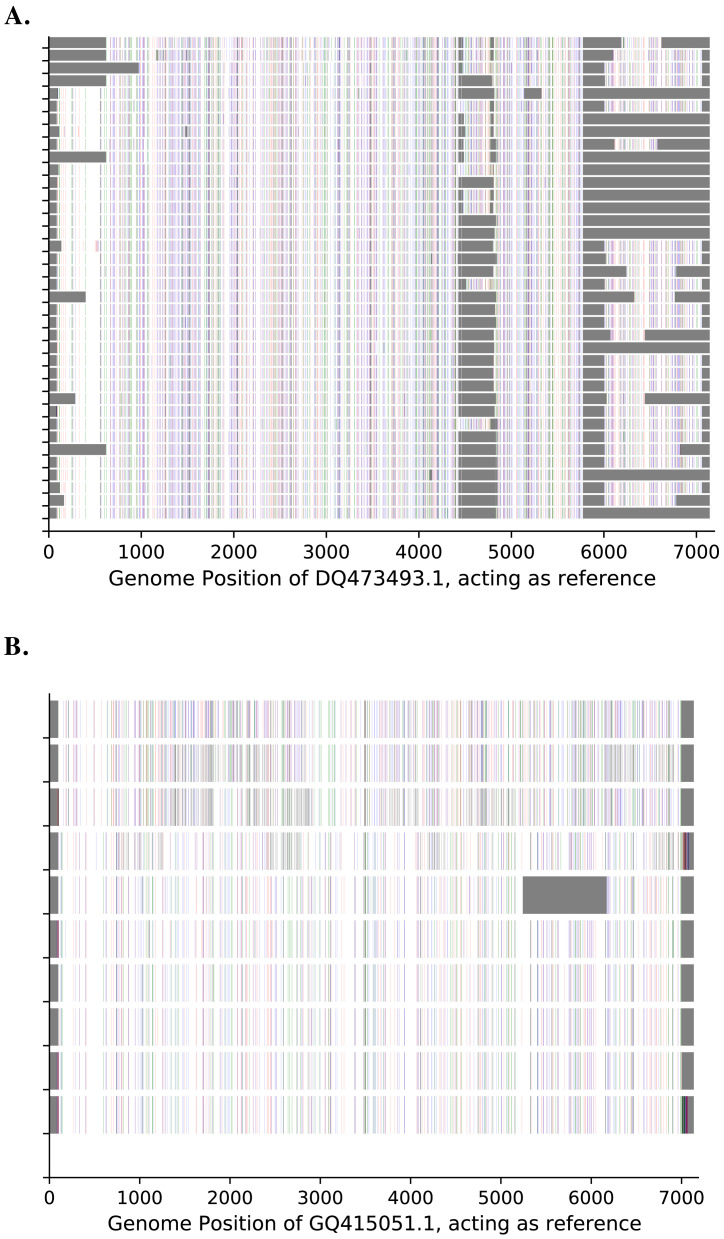
Genetic diversity across the genome for ( **A**) A15 and ( **B**) A101. The known HRV strains ( https://www.picornaviridae.com/sg3/enterovirus/rv-a/rv-a_seqs.htm) are used as the reference. A substitution to "A" is indicated by green, "C" by blue, "G" by indigo and "T" by red bars. Gray contiguous bars indicate unknown/unsequenced bases.

The ends of the 5' untranslated region (UTR) and 3' UTR were not amplified due to lack of suitable primers. Genetic diversity was observed across the entire genome and not within a particular genomic region for both types, as shown in Figure 3.

### Phylogenetic resolution

**Figure 4.**
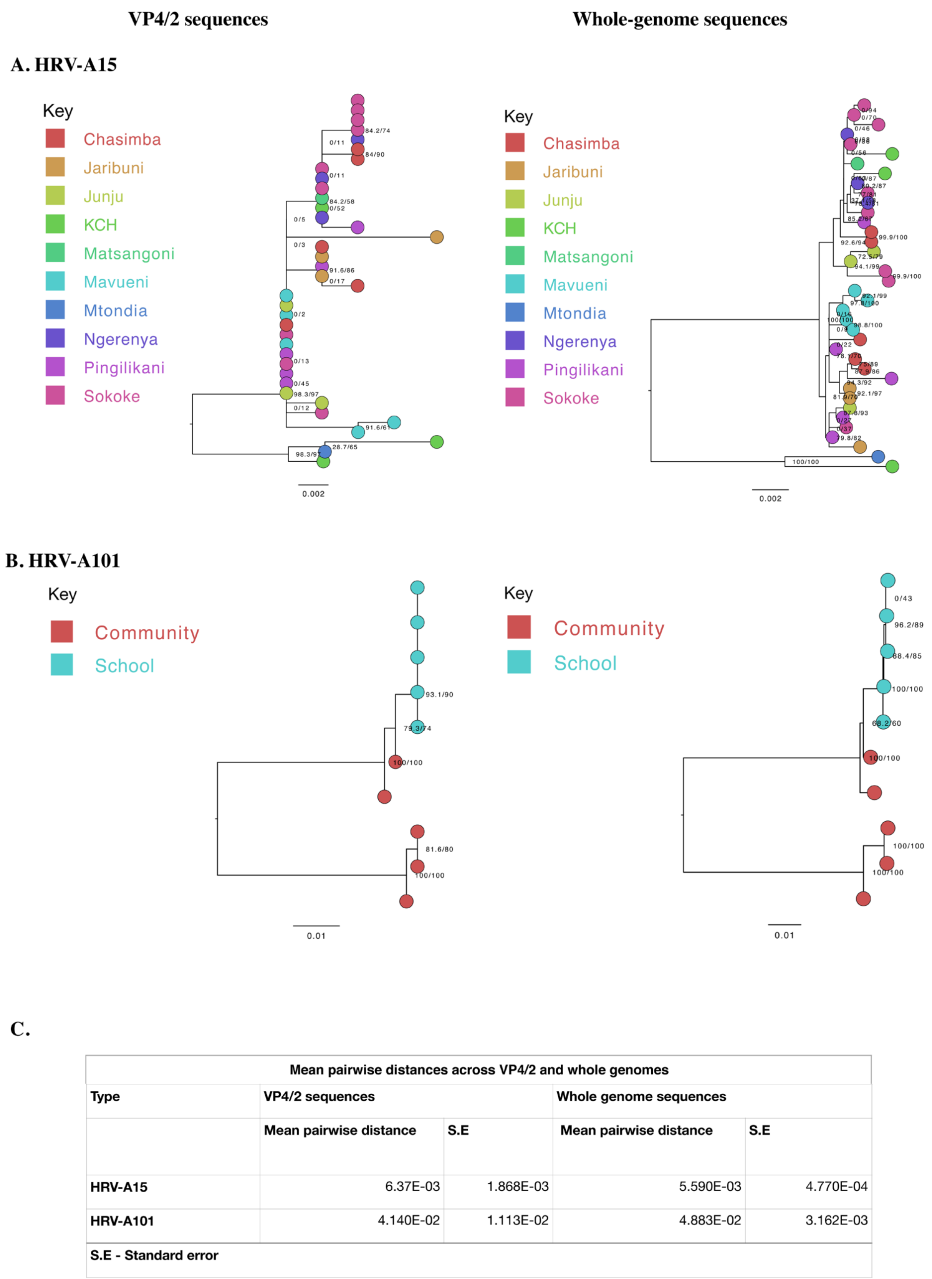
Maximum-likelihood phylogenetic trees of local ( **A**) A15 and ( **B**) A101 VP4/2 and whole genome sequences. The tips are coloured by site of origin. The scale bar represents nucleotide substitutions per site while node labels indicate bootstrap value. ( **C**) Mean pairwise distances and respective standard errors of VP4/2 and whole genome sequences.

We compared the phylogenetic bifurcation patterns and statistical uncertainty of VP4/2 and WGS for the two types. The depiction of sister taxa was comparable across the two trees. However, WGS resolved phylogenetic polytomies/unresolved branches observed previously in the VP4/2 phylogenies. For example, using VP4/2 sequences, all viruses collected in the school formed one polytomy, which was now fully bifurcated using WGS. Similarly, for A15, the four polytomies observed on VP4/2 phylogeny were well resolved using WGS, effectively distinguishing one sample from the other. Although the mean pairwise distances across VP4/2 and WGS were close, the standard error of pairwise distance calculations was notably less (about a tenth) in WGS than VP4/2, Figure 4.

**Figure 5.**
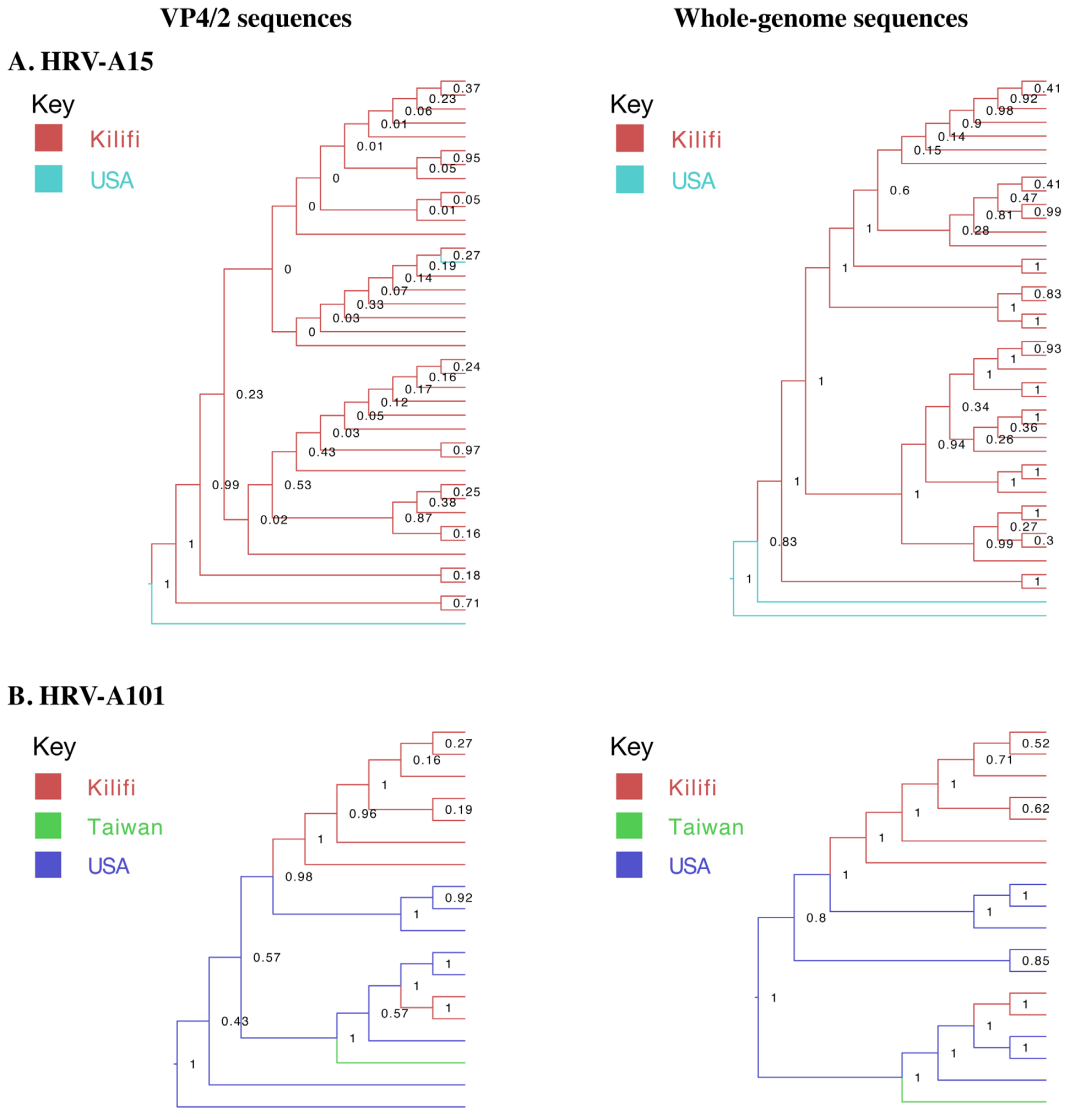
Bayesian phylogenetic trees of local and global ( **A**) HRV-A15 and ( **B**) HRV-A101 VP4/2 and whole genomes. The branches are coloured by site of origin. Node labels indicate branching posterior probabilities.

Overall higher branching posterior probabilities in Bayesian phylogenetic trees were observed using WGS than VP4/2 sequences. In VP4/2 Bayesian trees, 17.5% of A15 and 64.7% of A101 nodes had a posterior probability greater than 0.7 compared to 64.3% and 88.2% nodes in WGS trees, respectively, as illustrated in Figure 5.

**Figure 6.**
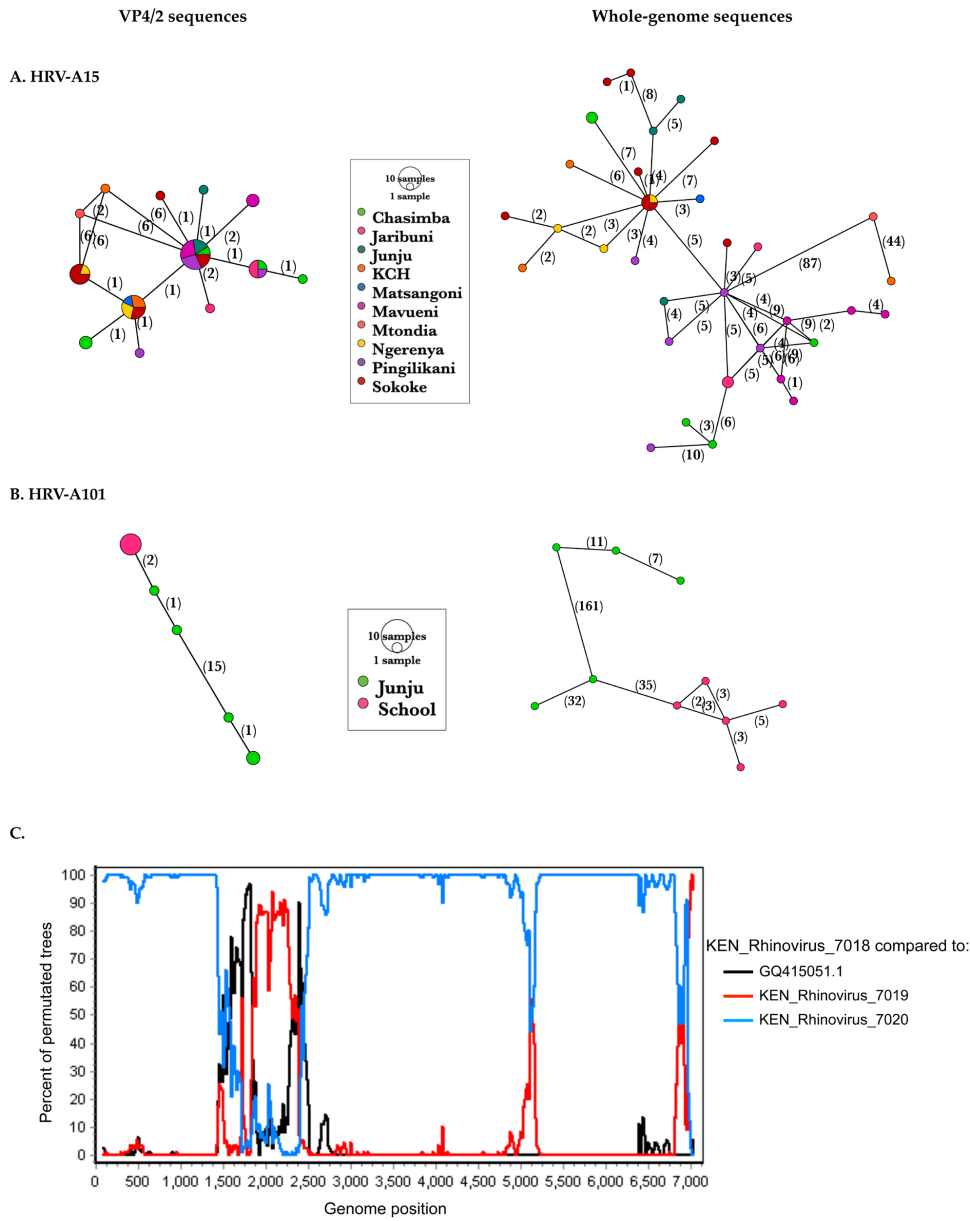
Haplotype networks displaying sequence variation of VP4/2 and whole-genome sequences of ( **A**) A15 and ( **B**) A101. Numbers along the edges indicate the nucleotide substitutions. The alleles are coloured by study site. ( **C**) Recombination scan of recombinant sequence KEN_Rhinovirus_7018 compared to its major and minor parents and the A101 prototype sequence, GQ415051.1. A putative recombinant region was identified within the VP3.

The improved resolution was further depicted by haplotype networks that displayed notably more alleles using WGS than VP4/2, e.g., in HRV-A101, school sequences that were considered a single allele using VP4/2 sequences resolved into five alleles when using whole-genome sequences, Figure 6. Identical samples at the VP4/2 region had a median of 3 nt changes for A101 and 5 nt changes for A15 across the whole genome. 

### Recombination analysis

Recombination scans identified breakpoints within the VP3 of one A101 sequence (KEN_Rhinovirus_7018), with both parents belonging to A101 type (p-value < 1.922E-2), Figure 6C. Recombination within HRV structural regions has been shown to be rare and sporadic ^30^.

## Discussion

This study presents a type-specific whole genome sequencing protocol for two HRV types. A101 had a higher success (100%) than A15 (60.3%). We attribute the higher A101 sequencing success to the higher number of sequences (n=9) that were available for primer design, which captured more intra-type variation, compared to A15 (n=3). Having more genomes contributing to the consensus sequence used in primer design increased genetic variation, and subsequently, the likelihood that the local and contemporaneous diversity was captured.

Whole-genome sequencing provided greater phylogenetic resolution and less statistical uncertainty to partial sequencing. Polytomies are a product of inadequate data and are, therefore, a potential source of bias. They also result in reduced statistical power due to increased uncertainty. The loss of terminal phylogenetic resolution may result in two opposing predictions: the underestimation (due to unresolved taxa) or overestimation (due to increased total tree length) of diversity ^31^. Due to the short size of VP4/2 (~420nt), insufficient data results in reduced phylogenetic resolution and increased uncertainty evidenced by a higher standard error in phylogenetic distance. Unresolved phylogenies are a challenge in epidemiology as one cannot distinguish infections from one individual to another for transmission inference.

Posterior probabilities summarize the uncertainties about a parameter and indicate confidence in the evidence ^32^. High posterior probabilities indicate high confidence, and the reverse is also true. Whole genomes consistently provided higher confidence across the two genotypes assessed in this study.

With pathogen sequencing now an established tool to track viral infections ^2, 12^, it is crucial to compare the resolution of different sequence analysis. As the huge antigenic diversity of HRV continues to pose a challenge in vaccine development, efforts should be directed towards understanding and mitigating transmission. Our study shows that HRV WGS is better suited for transmission inference to the commonly used VP4/2 sequences.

The sequencing approach we developed has some limitations. First, it requires prior genotyping of the HRV positive samples, commonly done by VP4/2 or VP1 sequencing. It is therefore unsuitable for sequencing new or highly divergent types due to the requirement of matching primer sequencing. Second, having to create primer sets for each type is cumbersome and relies on adequate number of pre-existing genomes to design conserved primers. In addition, an amplicon-based target enrichment does not work well for low complexity regions such as the 5'UTR and 3' UTR. Notwithstanding, the new method can successfully enrich for human rhinovirus in archived samples of varying virus titers. It can also effectively capture intra-type recombinant regions enabling detailed study of viral dynamics.

## Conclusions

With HRV being the most common respiratory virus, it is surprising that we have such few publicly available whole genomes to allow detailed intra-type analysis. We describe a new protocol for the whole genome sequencing of two HRV types and enrich the public database of HRV genomes. The protocol can be adapted for other HRV types. Our study also shows that WGS is more informative than VP4/2 sequencing in studying HRV dynamics as it maximizes resolution and reduces phylogenetic uncertainty.

## Data availability

Accession number: GenBank, MW713746-MW713793

Accession number: BioProject, PRJNA701406

Root URL: https://identifiers.org/bioproject

Accession number URL: https://identifiers.org/bioproject:PRJNA701406

Harvard Dataverse. Replication Data for: Whole genome sequencing of two human rhinovirus A types (A101 and A15) detected in Kenya, 2016–2018. DOI: https://doi.org/10.7910/DVN/QGXZLI ^33^

This project contains the following underlying data:

- This is a replication dataset for the manuscript titled: "Whole genome sequencing of two human rhinovirus A types (A101 and A15) detected in Kenya, 2016–2018." The dataset contains contains Cycle threshold (Ct) values, and read/sequencing depth.

Data are available under the terms of the Creative Commons Attribution 4.0 International license (CC-BY 4.0).
