# Supplementary material for: Whole genome sequencing of two human rhinovirus A types (A101 and A15) detected in Kenya, 2016-2018
Source: Wellcome Open Res. 2021 Sep 23;6:178. Originally published 2021 Jul 8. [Version 2] doi: 10.12688/wellcomeopenres.16911.2 (PMC8408540; doi:10.12688/wellcomeopenres.16911.2)
Supplement: Peer review history

# Peer review: Reviewer response for version 1

**DOI:** 10.21956/wellcomeopenres.18656.r45239

**Published:** 2021-08-31

**Authors:** Yewande Nejo

**Affiliations:** 1 Microbiology Programme, College of Agriculture, Engineering and Science, Bowen University, Iwo, Nigeria

**Overall comments:**

 This is an important and well executed study by Luka *et al.* (2021). It provides detailed informative data on the type-specific whole genome sequencing method designed to detect human rhinovirus. The study is able to present target enrichment sequencing approach which addresses the hurdles in genome amplification and sequencing of human rhinovirus posed by the high genetic diversity of the virus and inadequate information of reference sequences. The study is properly designed; however, it will require few additions.

**Abstract:**

 The abstract is a good summary of the research article but please indicate in the abstract the type of samples that were collected.

**Introduction:**

 The introduction provides information that is significant to the research and is well referenced. The importance and purpose of the study is well explained and justifiable.

**Methodology:**

 The methodology segment is well designed and all analyses were carried out with details and sound scientific merit. The author should however specify in this section the type of research carried out.

**Results:**

 The results are well stated and illustrated in a comprehensive manner. The data is meticulously analyzed

**Discussion and Conclusion:**

 The results of this study are well discussed and the data supports the conclusion. The limitations of the study are well stated. However, the reasons for selecting only two types of HRV for whole genome sequencing out of 12 types were not properly indicated. Can the author also explain what could be responsible for the sequencing failure of 25 samples? What could be the implications of not sequencing 5’UTR and 3’ UTR regions in the study of viral dynamics?

Is the work clearly and accurately presented and does it cite the current literature?

Yes

If applicable, is the statistical analysis and its interpretation appropriate?

Yes

Are all the source data underlying the results available to ensure full reproducibility?

Yes

Is the study design appropriate and is the work technically sound?

Yes

Are the conclusions drawn adequately supported by the results?

Yes

Are sufficient details of methods and analysis provided to allow replication by others?

Yes

Reviewer Expertise:

Virology

I confirm that I have read this submission and believe that I have an appropriate level of expertise to confirm that it is of an acceptable scientific standard.

# Response

**Published:** 2021-09-21

**Authors:** Martha Luka

**Affiliations:** KEMRI-Wellcome Trust Research Programme, Kenya

Thank you for taking the time to review our article. Please see below our responses to the issues raised.

**Abstract**

**Please indicate in the abstract the type of samples that were collected.**

 Nasopharyngeal swabs. (This has now been specified in the abstract)

**Methodology**

**The author should specify in this section the type of research carried out.**

 We used a combination of genomic, method-development, descriptive and retrospective approaches to develop a new laboratory protocol for the whole-genome sequencing of human rhinovirus and compare the phylogenetic inferences between partial and whole genome sequences

**Discussion and Conclusion**

**The reasons for selecting only two types of HRV for whole genome sequencing out of 12 types were not properly indicated.**

 The current study was nested within a larger epidemiological study to study pathways of respiratory virus disease within different scales which included: (i) outpatient surveillance of nine health dispensaries within the Kilifi Health and Demographic Surveillance System (KHDSS)[1] and (ii) a primary school surveillance[2]. The most frequent type observed in the KHDSS health dispensary surveillance was A15 (n=63). The second social scale, the school, was situated in Junju, a location within the KHDSS. Comparison of the HRV diversity within the school and the Junju outpatient clinic revealed 12 common types[3], and the most frequent common type was A101 (n=5 in each setting). These two types were identified as of interest due to their high frequency of occurrence at the various scales of observation studied.

**Can the author also explain what could be responsible for the sequencing failure of 25 samples?**

 While it’s not clear what the cause of sequencing failure of the 25 samples was, we speculate that either (i) their genomic diversity was not captured in primer design resulting in primer mismatches, (ii) the sample quality had deteriorated over time or (iii) there was presence of PCR inhibitors/ nuclease enzymes in the sample.

**What could be the implications of not sequencing 5’UTR and 3’ UTR regions in the study of viral dynamics?**

 Analysis of the 5’UTR across HRV shows high conservation of certain regions[4], making it a suitable target for HRV detection assays[5]. Contrastingly, the ~50 bp long 3’UTR is not universally conserved across HRV species[4]. Although the 5’UTR alone has not been proved to offer adequate resolution to confidently distinguish HRV types[6], it is speculated to be a hotspot for recombination[7]. Not sequencing these extreme regions may therefore result in missing out on important evolutionary/phylogenetic signal.

**References**

 1.        Nyiro JU, Munywoki P, Kamau E, et al. Surveillance of respiratory viruses in the outpatient setting in rural coastal Kenya: baseline epidemiological observations. Wellcome open Res. 2018;3:89.

 2.        Adema IW, Kamau E, Uchi Nyiro J, Otieno GP, Lewa C, Munywoki PK, Nokes DJ. Surveillance of respiratory viruses among children attending a primary school in rural coastal Kenya [version 1; peer review: awaiting peer review]. Wellcome Open Res [Internet]. 2020;5(63). Available from: https://wellcomeopenresearch.org/articles/5-63/v1

 3.        Luka MM, Kamau E, Adema I, et al. Molecular epidemiology of human rhinovirus from one-year surveillance within a school setting in rural coastal Kenya. medRxiv [Internet]. 2020 Jan 1;2020.03.09.20033019. Available from: http://medrxiv.org/content/early/2020/03/19/2020.03.09.20033019.abstract

 4.        Kloc A, Rai DK, Rieder E. The Roles of Picornavirus Untranslated Regions in Infection and Innate Immunity. Front Microbiol. 2018;9:485.

 5.        Hammitt LL, Kazungu S, Welch S, et al. Added Value of an Oropharyngeal Swab in Detection of Viruses in Children Hospitalized with Lower Respiratory Tract Infection. J Clin Microbiol [Internet]. 2011 Jun 1;49(6):2318 LP – 2320. Available from: http://jcm.asm.org/content/49/6/2318.abstract

 6.        Savolainen-Kopra C, Blomqvist S, Smura T, et al. 5’ noncoding region alone does not unequivocally determine genetic type of human  rhinovirus strains. Vol. 47, Journal of clinical microbiology. United States; 2009. p. 1278–80.

 7.        McIntyre CL, Savolainen-Kopra C, Hovi T, Simmonds P. Recombination in the evolution of human rhinovirus genomes. Arch Virol. 2013;158(7):1497–515.

# Peer review: Reviewer response for version 1

**DOI:** 10.21956/wellcomeopenres.18656.r45240

**Published:** 2021-08-17

**Authors:** Martin Munene Nyaga

**Affiliations:** 1 South African Medical Research Council/Diarrhoeal Pathogens Research Unit, Faculty of Health Sciences, Sefako Makgatho Health Sciences University, Pretoria, South Africa

**Overall comments:**

 Luka *et al. *(2021) present a type-specific full genome sequencing approach for obtaining genomic data of human rhinovirus. The authors have done an excellent job of addressing a research gap in rhinovirus amplification and sequencing challenges caused by a high genetic diversity and relatively inadequate reference sequences. The study is well designed, with detailed analysis and well-reported conclusions.

**Abstract:**

 The abstract concisely summarizes the research article.

**Introduction:**

 The introduction is well written. The authors have provided a sufficient background and articulated the purpose of the research work in a concise manner.

**Methodology:**

 The methodology section is well designed.

**Results:**

 The result findings are well described. The data analysis is very thorough.

**Discussion and conclusion:**

 The discussion part is well-written and the authors have done a good job of describing the study’s shortcomings. It is commendable to see that, despite the limitations, the study’s findings corroborate the type-specific sequencing approach being promoted. A question that may need more discussion on is: what could have caused the sequencing failure of 25 samples with a median ct value that was not significantly higher than those successfully sequenced? The conclusion wraps up the study nicely.

Is the work clearly and accurately presented and does it cite the current literature?

Yes

If applicable, is the statistical analysis and its interpretation appropriate?

I cannot comment. A qualified statistician is required.

Are all the source data underlying the results available to ensure full reproducibility?

Yes

Is the study design appropriate and is the work technically sound?

Yes

Are the conclusions drawn adequately supported by the results?

Yes

Are sufficient details of methods and analysis provided to allow replication by others?

Yes

Reviewer Expertise:

Virology (eneteric and respiratory )

I confirm that I have read this submission and believe that I have an appropriate level of expertise to confirm that it is of an acceptable scientific standard.

# Response

**Published:** 2021-09-20

**Authors:** Martha Luka

**Affiliations:** KEMRI-Wellcome Trust Research Programme, Kenya

Thank you for taking the time to review our manuscript. Please find below our response (in *italics*) to the issue raised .

 A question that may need more discussion on is: what could have caused the sequencing failure of 25 samples with a median Ct value that was not significantly higher than those successfully sequenced?

*While it’s not clear what the cause of sequencing failure of the 25 samples was, we speculate that either (i) their genomic diversity was not captured in primer design resulting in primer mismatches, (ii) the sample quality had deteriorated over time or (iii) there was presence of PCR inhibitors/ nuclease enzymes in the sample.*
